# Supplementary material for: Poorer Survival in Patients with Cecum Cancer Compared with Sigmoid Colon Cancer
Source: Medicina (Kaunas). 2022 Dec 27;59(1):45. doi: 10.3390/medicina59010045 (PMC9864791; doi:10.3390/medicina59010045)
Supplement: Supplementary file 1 [file medicina-59-00045-s001.zip › medicina-2055396-supplementary.pdf]

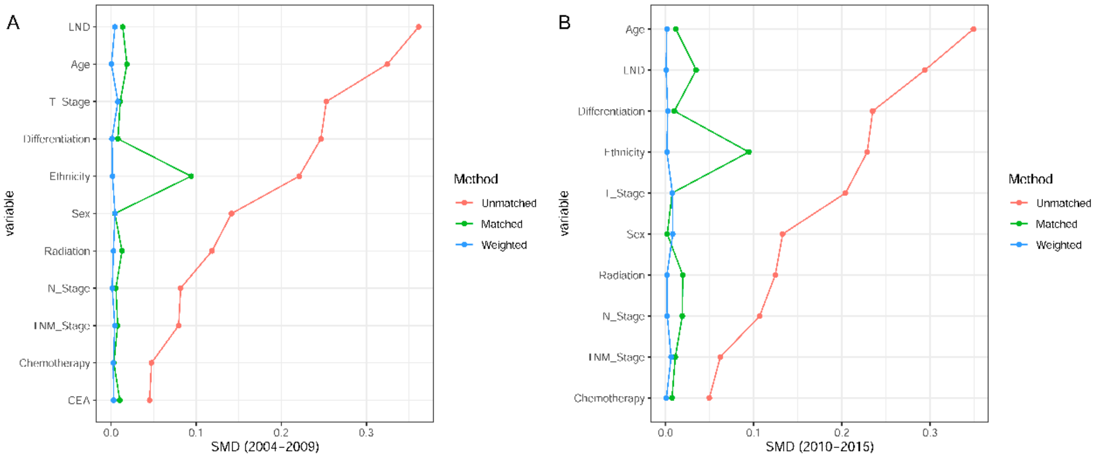

Figure S1. Comparison of unmatched, matched and weighted data. (**A**) 2004–2009. (**B**) 2010–2015.

Table S1. Demographic data for patients with colon cancer in SEER database from 2004 to 2009 and 2010 to 2015.

Characteristic	2004–2009	2010–2015	P-value
No. subjects (%)	45,645 (49.6)	46,360 (50.4)	
Median age at diagnosis (years)	65	64	
Sex			
Female	22,654 (49.6)	22,486 (48.5)	
Male	22,991 (50.4)	23,874 (51.5)	< 0.001
Ethnicity			
Caucasian	35,556 (77.9)	35,237 (76.0)	
African-American	5,849 (12.8)	6,221 (13.4)	
Others/Unknown	4,240 (9.3)	4,902 (10.6)	< 0.001
Tumor location			
Cecum	16,313 (35.7)	16,175 (34.9)	
Ascending colon	3,308 (7.2)	3,568 (7.7)	
Hepatic flexure	1,961 (4.3)	1,798 (3.9)	
Splenic flexure	2,637 (5.8)	2,348 (5.1)	
Descending colon	9,935 (21.8)	10,941 (23.6)	
Sigmoid colon	11,491 (25.2)	11,530 (24.9)	< 0.001
Differentiation			
Well	4,539 (9.9)	4,244 (9.1)	
Moderately	33,088 (72.5)	34,553 (74.5)	
Poorly	7,434 (16.3)	6,348 (13.7)	
Undifferentiated	584 (1.3)	1,215 (2.6)	< 0.001
LND			
< 12	13,669 (29.9)	5,683 (12.3)	
≥ 12	31,976 (70.1)	40,677 (87.7)	< 0.001
LNM			
No	28,256 (61.9)	29,140 (62.9)	

Yes	17,389 (38.1)	17,220 (37.1)	0.003
AJCC T-stage			
T1	6,550 (14.3)	7,251 (15.6)	
T2	7,645 (16.7)	7,667 (16.5)	
T3	26,444 (57.9)	24,895 (53.7)	
T4	5,006 (11.0)	6,547 (14.1)	< 0.001
AJCC N-stage			
N0	28,130 (61.6)	28,389 (61.2)	
N1[a]	11,219 (24.6)	11,884 (25.6)	
N2	6,296 (13.8)	6,087 (13.1)	< 0.001
AJCC TNM stage			
I	11,832 (25.9)	12,409 (26.8)	
II	16,298 (35.7)	15,980 (34.5)	
III	17,515 (38.4)	17,971 (38.8)	< 0.001
CEA			
Negative	16,968 (37.2)	18,052 (38.9)	
Positive	8,515 (18.7)	9,280 (20.0)	
Borderline	187 (0.4)	157 (0.3)	
Others	19,975 (43.8)	18,871 (40.7)	< 0.001
Radiation therapy			
No	44,753 (98.0)	45,663 (98.5)	
Yes	892 (2.0)	697 (1.5)	< 0.001
Chemotherapy			
No/unknown	30,027 (65.8)	30,119 (65.0)	
Yes	15,618 (34.2)	16,241 (35.0)	0.009

Notes: [a] N1 included tumor deposits besides lymph node metastasis; **Abbreviations:** SEER the Surveillance, Epidemiology, and End Results; LND lymph node dissection; LNM lymph node metastasis; AJCC American Joint Committee on Cancer; T primary tumor; N regional lymph nodes; M distant metastasis; CEA carcinoembryonic antigen.
